# Damage Orientation and Depth Effect on the Guided Wave Propagation Behavior in 30CrMo Steel Curved Plates

**DOI:** 10.3390/s20030849

**Published:** 2020-02-05

**Authors:** Chaojie Hu, Bin Yang, Fu-Zhen Xuan, Jianjun Yan, Yanxun Xiang

**Affiliations:** School of Mechanical and Power Engineering, East China University of Science and Technology, No. 130 Meilong Road, Shanghai 200237, China; y20170063@mail.ecust.edu.cn (C.H.); jjyan@ecust.edu.cn (J.Y.); yxxiang@ecust.edu.cn (Y.X.)

**Keywords:** curved plates, guided waves, notch localization, structural health monitoring, pressure vessels

## Abstract

In this paper, the guided wave propagation behavior in damaged 30CrMo steel curved plates was investigated experimentally and numerically. The effects of the notch orientation, depth in the curved plate, as well as its radius, on the wave propagation characteristics were mainly analyzed by the amplitude distribution curves and the directivity diagrams of A0/S0 (zero-th order of the symmetric/antisymmetric Lamb wave) modes. An ellipse-based algorithm was compiled to locate the notches in the curved plates. Results show that the normalized S0 wave amplitude in the circumferential orientation was the largest, and it increases as notch depth increases in the axial orientation. The A0 wave amplitude in axial orientation was the largest, while it decreases with the increasing of notch depth in the other orientations. The normalized A0 wave amplitude in axial orientation increases with the increasing of radius. With the increasing of radius, the other normalized A0/S0 amplitudes linearly decreased for the other paths. The ellipse-based algorithm has high notch localization accuracy, and the notch localization error increase from 0.005% to 1.47% with the notch depth decreasing from 5 mm to 1 mm in the curved plates. For the curved plates with different radius, the maximum notch localization error is 1.20%. These satisfactory results demonstrate the effectiveness of the developed algorithm in locating damages in the researched structure.

## 1. Introduction

Pressure vessels are commonly used in the petrochemical, space engineering, nuclear industries etc. [1]. These pieces of equipment are usually operated under extremely high pressures and high/low temperatures, and the harsh service environment may lead to leakage or rupture failures [2,3,4], which can cause considerable loss to life and property [5]. Cylindrical pressure vessels are currently the most widely used pressure vessels, and their failure often occurs in the cylindrical section [6]. Therefore, it’s necessary to be able to determine the health status of the cylindrical section in pressure vessels.

Recently, structural health monitoring (SHM) technology is attracting increasing interest from researchers [7]. By checking the safety status continuously or as needed, it can quickly capture the overall structural health state [8]. Moreover, SHM technology can reduce the inspection costs of in-service equipment [9]. At present, this technology has been successfully applied in the Sutong Bridge [10], Runyang Bridge [11], and Boeing 737 aircraft [12], etc. Compared with other SHM technologies, ultrasonic guided waves can quickly interrogate large structures and are sensitive to both surface and subsurface features [13]. Therefore, guided waves have shown great advantages in various applications as a SHM solution [8]. In terms of health monitoring of pressure vessels, this active monitoring method has great application prospects to effectively determine the damage location, severity and types [2]. Although numerous studies have been conducted in SHM field, a systematic understanding of guided waves in pressure vessels for SHM purpose is lacking, not to mention the gap that still exists between laboratory successes and real industrial applications.

A large number of references have reported the guided waves propagation behavior in hollow cylinders and/or plates [14,15,16,17,18]. For instances, Singh et al. [15] developed a parametric 2D finite-element technique, which can size strip-like defects in elastic or viscoelastic, isotropic or anisotropic plates using guided waves. A non-destructive, ultrasonic guided wave technique was presented by Crom et al. [18] to evaluate the bonding quality between substrates. Fletcher et al. [16] investigated the implementation of synthetic guided wave focusing to locate axially aligned defects in hollow cylinders. Non-axisymmetric modes of guided waves were used for damage detection in hollow cylinders after appropriate mode tuning under certain circumstances by Gavigan et al. [17]. The work of Yu et al. [19] shows that the radius to thickness ratio has significant influences on the wave characteristics for graded spherical curved plates. All the mentioned works found that the interaction between damages and guided waves exhibit complex phenomena, such as dispersion, multiple modes, damage interaction, and mode conversion etc. [14]. However, to the best of our knowledge, there are few studies using guided wave-based SHM techniques to locate the damage in large and complex structures such as pressure vessels. Ultrasonic guided wave modes are proposed by Castaings et al. [20] to test the moisture content and the micro-cracking of carbon-epoxy composite wound pressure vessels. Li et al. [2] studied the guided wave propagation characteristics in the pressure vessels by a combination of analytical method, finite element analysis and experiments. In our previous work, we used the coordinate transformation method to locate the damage position in pressure vessel [21]. The results indicate that the propagation of guided waves in pressure vessels is very complex.

Since the cylinder section of a pressure vessel is mainly comprised of curved plates, therefore according to the geometric similarity, 30CrMo steel curved plates were adopted in this paper. This material is an alloy structural steel, which is a common raw material for vehicle pressure vessels [22]. Signal acquisition and processing system were established to obtain the guided wave signals, and STFT (the short-time Fourier transform) was adopted to verify the experimental and simulation results. The normalized S0/A0 wave amplitudes were extracted to determine the relationships between the wave features and notch depths, orientations and the curved plate radius. Finally, an ellipse-based algorithm was compiled to locate the notches in the curved plates.

## 2. Theoretical Background

### 2.1. Guided Wave Propagation in Curved Plate

**Figure 1:**
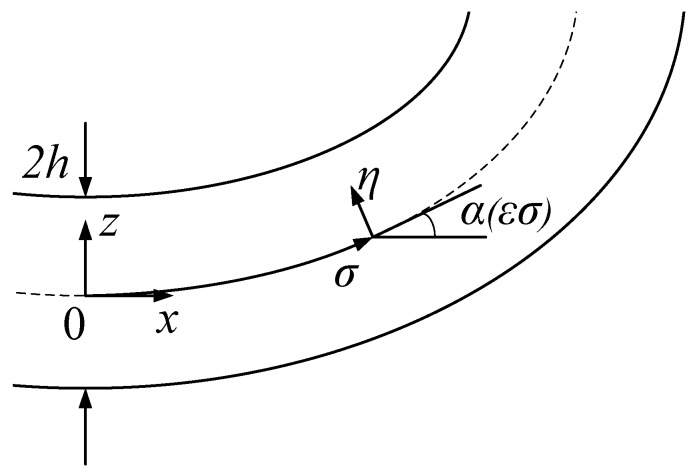
The homogeneous and isotropic linearly elastic curved plate.

We consider a homogeneous and isotropic linearly elastic curved plate with constant thickness of 2 h, as shown in Figure 1. The density of the material is ρ, and its Lame’ constants are λ and μ, respectively.

An orthogonal curvilinear coordinate (*σ*, *η*) is adopted, where *σ* is the arc-length along the centerline, and *η* is the shortest distance from the observation point to the centerline of the guided wave. The angle *α* is between a tangent to the centerline and x-axis, which is a smooth function *ξ* = *εσ* (*α* = *α*(*ξ*), *ε* is a small dimensionless parameter). The parameter *ε* can be assumed to the ratio of *h* to a typical radius. The time-harmonic wave propagation was studied in the layer, and the common factor exp(−*iωt*) (*ω* is the angular frequency) will be understood and is henceforth suppressed. The displacement vector *u* can be represented of two scalar potentials *φ* and *ψ* in the Cartesian coordinate system as follows [23]:

(1) $$u=(\phi_{x}+\psi_{x},\phi_{z}-\psi_{x})$$

where the subscripts represent the partial derivatives with respect to the corresponding variables, and the potentials satisfy the Helmholtz equations:

(2) $$u=(\phi_{x}+\psi_{x},\phi_{z}-\psi_{x})$$

where *k_L_* = *ω*/*c_L_* and *k_T_* = *ω*/*c_T_* are the bulk longitudinal and transverse wave numbers, and *C_L_* = [(*λ* + 2*μ*)/*ρ*]^½^ and *C_T_* = (*μ*/*ρ*)^½^ are the longitudinal and shear wave velocities, respectively.

Equation (2) is transformed into:

(3) $$\begin{array}{l} \kappa^{2}\phi_{\sigma \sigma }+\phi_{\eta \eta }+\kappa^{3}\alpha_{\sigma \sigma }\eta \phi_{\sigma }-\kappa \alpha_{\sigma }\phi_{\eta }+k_{L}^{2}\phi =0\\ \kappa^{2}\psi_{\sigma \sigma }+\psi_{\eta \eta }+\kappa^{3}\alpha_{\sigma \sigma }\eta \psi_{\sigma }-\kappa \alpha_{\sigma }\psi_{\eta }+k_{L}^{2}\psi =0 \end{array}$$

A shorthand notation *κ* = (1 − *α_σ_η*)^−1^ is adopted here. Thus the displacement components *u* and *v* along the *σ* and *η* directions can be represented as follows:

(4) $$u=\kappa \phi_{\sigma }+\psi_{\eta },\;v=\phi_{\eta }-\kappa \psi_{\sigma }$$

The stress tensors are as follows:

(5) $$\begin{array}{l} \tau_{\sigma \sigma }=\mu (-k_{T}^{2}\phi -2\phi_{\eta \eta }+2\kappa \psi_{\eta \sigma }+2\kappa^{2}\alpha_{\sigma }\psi_{\sigma })\\ \tau_{\sigma \eta }=\mu (2\kappa \phi_{\eta \sigma }+2\kappa^{2}\alpha_{\sigma }\phi_{\sigma }+k_{T}^{2}\psi +2\psi_{\eta \eta })\\ \tau_{\eta \eta }=\mu \left[(2k_{L}^{2}-k_{T}^{2})\phi +2\phi_{\eta \eta }-2\kappa \psi_{\eta \sigma }-2\kappa^{2}\alpha_{\sigma }\psi_{\sigma }\right] \end{array}$$

Dimensionless variables $\bar{\xi =\xi /h}$ and $\bar{\eta }=\eta /h$ are considered. Finally, the following asymptotic approximation of a Lamb quasi-mode of curved plate can be obtained (the detailed information of the derivation process can be found in [23]):

(6) $$\begin{array}{l} \phi (\bar{\xi },\bar{\eta })≃(A^{\left(0\right)}+\varepsilon A^{\left(1\right)})\exp\left[\frac{i}{\varepsilon }k_{l}\bar{\xi }+\varepsilon^{2}s_{2}\int_{0}^{\bar{\xi }}\alpha_{\bar{\xi }}^{2}d\bar{\xi }\right]\\ \psi (\bar{\xi },\bar{\eta })≃i(B^{\left(0\right)}+\varepsilon B^{\left(1\right)})\exp\left[\frac{i}{\varepsilon }k_{l}\bar{\xi }+\varepsilon^{2}s_{2}\int_{0}^{\bar{\xi }}\alpha_{\bar{\xi }}^{2}d\bar{\xi }\right] \end{array}$$

where the phase function $S=\sum_{i=0}^{\infty }\varepsilon^{i}S^{\left(i\right)}$ is real-valued, and the hierarchy of equations for the amplitudes $A=\sum_{i=0}^{\infty }\varepsilon^{i}A^{\left(i\right)}$ and $B=\sum_{i=0}^{\infty }\varepsilon^{i}B^{\left(i\right)}$ are in general complex-valued.

The asymptotic approximations of the displacement components *u* and *v* for the quasi-modes can be obtained by substituting Equation (6) into Equation (4), as follows:

(7) $$\begin{array}{l} u≃i\left[k_{l}A^{\left(0\right)}+B_{\bar{\eta }}^{\left(0\right)}+\varepsilon (k_{l}\alpha_{\bar{\xi }}A^{\left(0\right)}+k_{l}A^{\left(1\right)}+B_{\bar{\eta }}^{\left(1\right)})\right]\times \exp\left[\frac{i}{\varepsilon }\left(k_{l}\bar{\xi }+\varepsilon^{2}s_{2}\int_{0}^{\bar{\xi }}\alpha_{\bar{\xi }}{}^{2}d\bar{\xi }\right)\right]\\ v≃i\left[k_{l}B^{\left(0\right)}+A_{\bar{\eta }}^{\left(0\right)}+\varepsilon (k_{l}\alpha_{\bar{\xi }}B^{\left(0\right)}+k_{l}B^{\left(1\right)}+A_{\bar{\eta }}^{\left(1\right)})\right]\times \exp\left[\frac{i}{\varepsilon }\left(k_{l}\bar{\xi }+\varepsilon^{2}s_{2}\int_{0}^{\bar{\xi }}\alpha_{\bar{\xi }}{}^{2}d\bar{\xi }\right)\right] \end{array}$$

where terms of order *ε*^2^ and higher in the amplitudes have been neglected.

### 2.2. Dispersion Characteristics of Guided Waves in 30CrMo Steel Curved Plates

**Figure 2:**
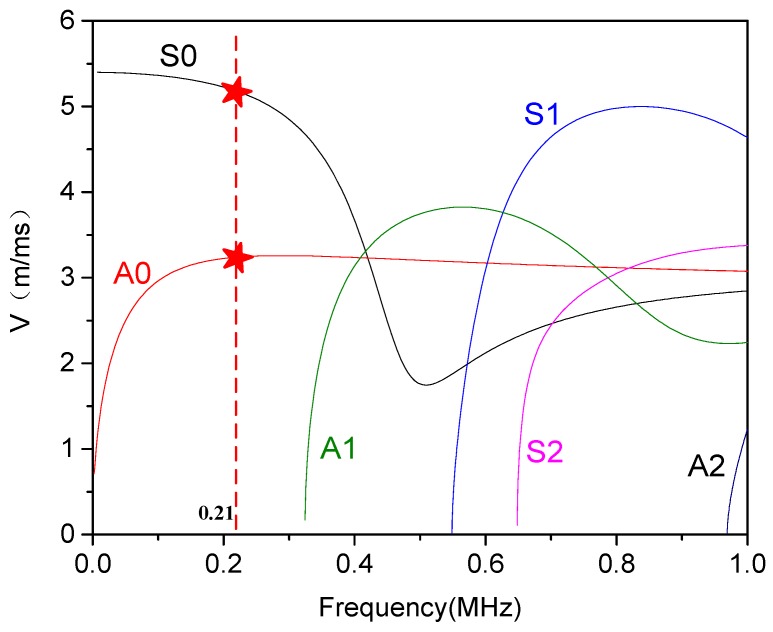
Dispersion curves of group velocity for a 5 mm length notch plate.

The thickness of the used 30CrMo steel curved plates is 5 mm, with a diameter-to-thickness ratio of 65. In this thin slab structure, guided waves are governed by the equations mentioned above with an infinite number of solutions, each of which represents a wave mode. The dispersion phenomenon of guided waves can be described by dispersion curves. The DISPERSE software was used to draw the group velocity of the 30CrMo steel curved plate, as shown in Figure 2 [24]. The multi-wave mode feature makes the propagation of guided waves very complex in the curved plates. Therefore, to simple the wave signal, we selected 0.21 MHz as the excitation frequency. As can be seen in the Figure 2, this frequency merely corresponds to two modes, and it further facilitates the mode analysis and separation work in the following signal processing.

### 2.3. Ellipse-Based Damage Localization Method

**Figure 3:**
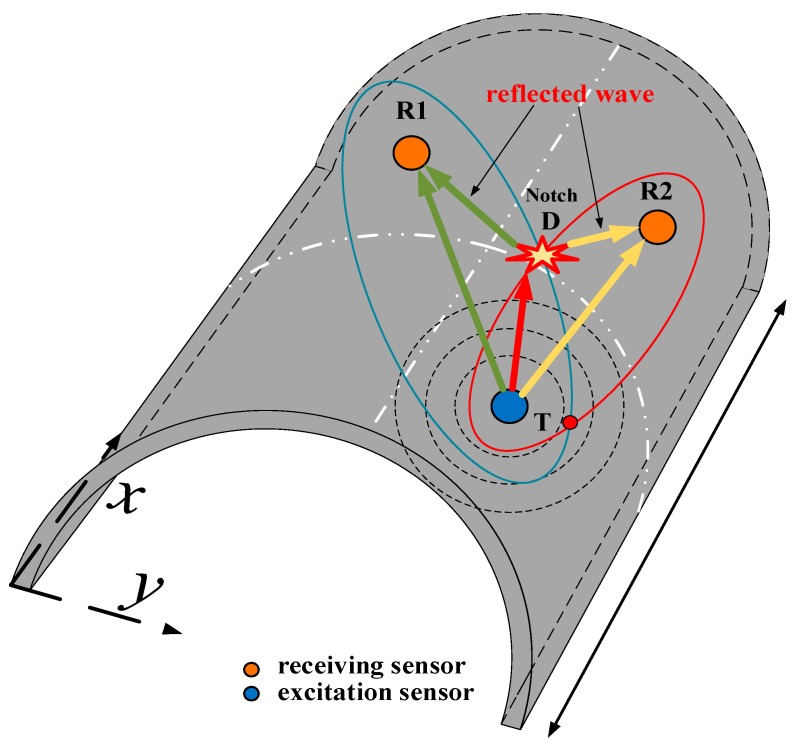
Schematic diagram of elliptical imaging method.

Figure 3 is the schematic diagram of the ellipse-based damage localization method. The sensor array and damage location were indicated in the figure, and *D*, *T* and *R* represent the notch, excitation and receiving sensor, respectively. The detailed information of the algorithm can be found in the [25]. Simply, Equation (8) was used to determine the damage location:

(8) $$S=TD+DR=c_{g}t$$

In the formula, *c_g_* is the group velocity of the guided wave signal and *t* is the propagation time of the scattered signal wave packet from the excitation sensor to the receiving sensor. The damage is located on the elliptic trajectory with *T* and *R* as the focus and *S* as the long axis.

An elliptical trajectory can be determined by any pair of sensors, and multiple elliptical trajectories can be determined by a multi-pair sensor network in the sensor array. The intersection of these elliptic trajectories is the location of the damage. In curved plates, the interactions between diverse modes and notches are different in various orientations. This suggests that the notch localization in the curved plate is different from that in the flat plate, and the *c_g_* corresponding to a certain mode cannot accurately locate the damage in curved plate independently. According to the interaction rules of guided wave modes and notches, the accuracy of notch localization can be improved by selecting different wave packets and corresponding wave velocities in different orientations. Accordingly, the notch orientation and depth effect on the propagation behavior of guided wave modes in curved plates were investigated, and an enhanced ellipse algorithm was introduced in the MATLAB environment to locate notch damage in curved plates.

## 3. Methodology

### 3.1. Online Structural Health Monitoring System

**Figure 4:**
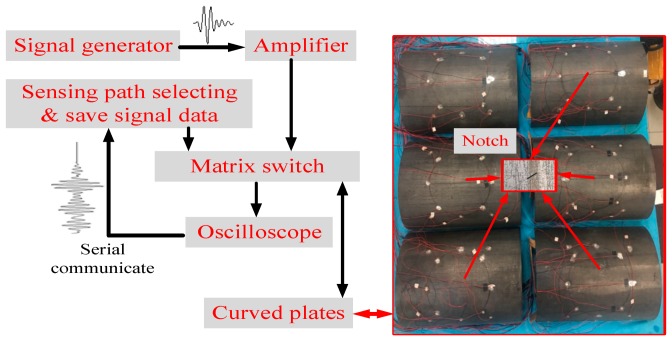
Hardware and software developed to real-time monitoring the curved plates.

An online structural health monitoring system was established to collect and processing guided wave signals in 30CrMo steel curved plates, as shown in Figure 4. In detail, the system contains the hardware and the corresponding driving software. The hardware is mainly composed of three components: the matrix switch of active sensor networks, multi-channel data acquisition, and a wave generation to generate the ultrasonic guided wave. The software is developed based on G language, and its main function is to control the hardware and process guided wave signals. The software and hardware exchange data through serial communicate. Piezoelectric ceramic transducers (PZTs) are automatically controlled by a 2 × 64 tunnel matrix switch, with any selected two PZTs acting as actuators and sensors simultaneously. The PZT formed a disc with 10 mm in diameter and 1 mm in thickness, and the material of PZT transducer is commercial PZT-PIC 151 ceramic with the chemical formula of Pb_0.92_Mg_0.04_Sr_0.025_Ba_0.015_(Zr_0.46_Ti_0.54_)O_3_, which can convert mutually mechanical energy and electrical energy. The vibration of the PZT is along its thickness direction, while the wave propagation expands along the radius direction. According to the results of sweep frequency, the center frequency of the transducer was obtained to be 210 kHz with 160 kHz–260 kHz bandwidth. All PZT transducers were bonded on the surface of the 30CrMo steel curved plates using cyanoacrylate adhesive. Lin et al. [26] showed that cyanoacrylate adhesive is more suitable for the attachment of PZT transducers in short term experiments compared to other adhesives. The Hamming-windowed 5 cycle pulse signal was excited by an AFG 3012C single channel arbitrary function generator (Tektronix, Beaver, OR, USA). Our previous work [21] showed that the five cycle pulse signal shows satisfactory positioning accuracy, hence the same signal was adopted in this paper. The signal was powered up to 40 W through the T&C power high-voltage amplifier (Model EPA-104), and then it was recorded/saved by the Tektronix MDO 3012 mixed domain oscilloscope and computer through the matrix switch, respectively.

### 3.2. Experimental and Simulation Method

**Figure 5:**
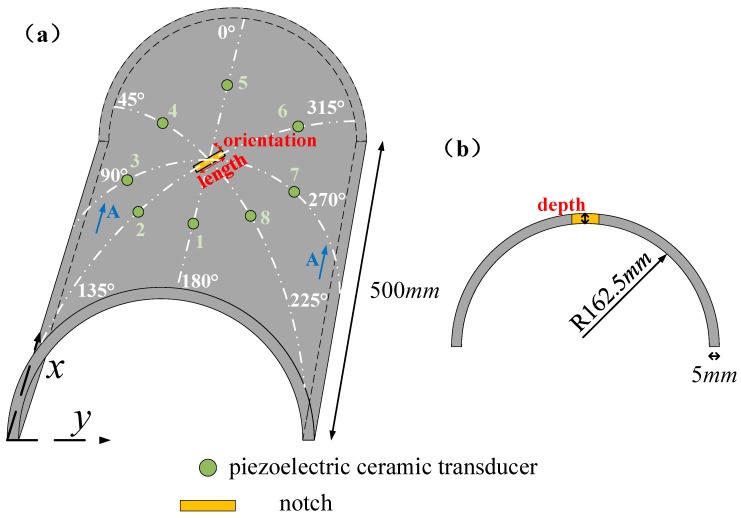
Schematic of the curved plate. (**a**) Isometric view and (**b**) front view.

As mentioned above, 30CrMo steel curved plates were adopted in the experiments. The dimension, notch form, and PZT array of the curve plate were schematically presented in Figure 5. The notch is located in the center of the curved plate with an angle of 45° relative to the axis direction of the structure. To study the notch depth effect on the guided waves propagation behavior, six notched plates with notch depths of 0 to 5 mm were prepared, and the curve plate with 0 mm notch was taken as the healthy sample. The sensor arrays with 0°, 45°, 90°, and 135° angles were arranged, and a total of 8 PZTs are numbered. In the following paper, the guide wave propagation path was coded as the excitation and reception sensor, such as 1–5, 2–6, 3–7, and 4–8. The 30CrMo steel curved plates used in the experiment were obtained through the procedures of rolling, heat and surface treatment.

The width of the notch was 0.8 mm and the length was 5 mm, which were manufactured by spark cutting. Guided wave signals from four orientations were considered in Figure 5. The results in each orientation were normalized against the maximum displacement amplitude in the incident wave. Since the center frequency of the transducer is 210 kHz with 160 kHz–260 kHz bandwidth, a band-pass filter with same range could reduce the noise outside the signal bandwidth [27]. Wavelet denoising process was carried out to obtain the waveform with high signal-to-noise ratio subsequently [28], and the signals obtained from the notched curved plates were subtracted from the notched plate without notches.

**Figure 6:**
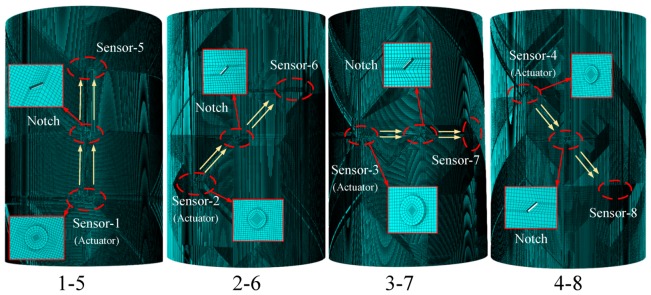
Finite element model of curved plates.

Finite element models were built in Abaqus software, and the models were in accordance with the curved plates in the experiment. The parameters of the 30CrMo steel curved plates are listed in Table 1. Six curved plate models with different notch depths were simulated, and the transducers were simulated to be a disc with 10 mm in diameter and 1 mm in thickness actuated by the Hamming-windowed five cycle pulse signal, the same as in the experiments. Four models were built for each curved plate, as shown in Figure 6. The explicit dynamic method was used to solve the elastic dynamic equation that controls the elastic guided wave propagation. Structured elements of the curved plate and PZT transducers were adopted, and C3D8R hexahedral solid element was selected [29]. In order to achieve stable solution, it must be ensured that eight elements per wavelength as follows [30]:

(9) $$\Delta x\leq \frac{\lambda_{\min}}{8}=\frac{c_{\min}}{8\times f}$$

where Δx is the length of the element, *λ*_min_ is the minimum wavelength of the guided wave, *c*_min_ is the minimum wave velocity of the guided wave, and *f* is the excitation frequency of the ultrasonic guided wave. According to the group velocity in the dispersion curves, the minimum wave velocity of the guided wave is 3200 m/s at 210 kHz, thus the mesh length of the element is:

(10) $$\Delta x\leq \frac{c_{\min}}{8\times f}=\frac{3200}{8\times 2.1\times 10^{5}}=1.9\times 10^{-3}m=1.9mm$$

In this paper, the mesh size of the model was selected as 1 mm.

The time increment has to be determined to ensure stability, and the time step should meet the following conditions [31]:

(11) $$T\leq \frac{D_{L}}{c_{g}}$$

where *D_L_* is the element size and *c_g_* is the longitudinal velocity. Therefore, the total calculation time can be set as 1 × 10^−7^ s.

## 4. Results and Discussion

### 4.1. Comparison between Experimental and Simulation Results

**Figure 7:**
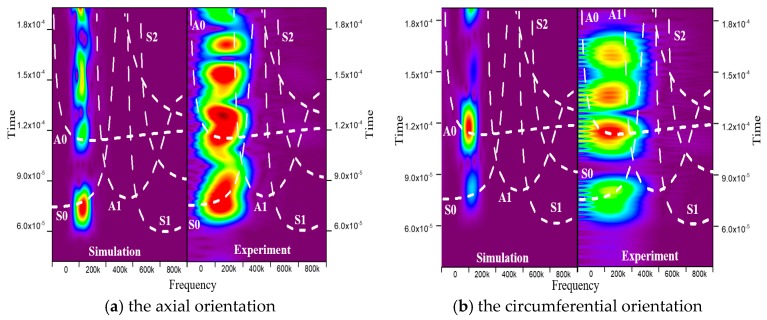
Time-frequency representation of the guided wave signals extracted from the simulation and experiment.

STFT is a mathematical transformation related to the Fourier transform to determine the frequency and phase of the wave in a local area of a time-varying signal [32]. This method was performed on the scatter wave signals, and the window size of STFT was carefully chosen such that sufficient details of both time and frequency information of the signal could be retained [33]. The acquired signals of 1–5 and 3–7 orientations in the Time-Frequency coordinate are shown in Figure 7, and the areas highlighted in the figure are the guided wave modes distribution over time. It can be seen that A0 and S0 modes were well separated, and the experimental and simulation results of the two modes were relatively consistent in the time domain at the frequency of 210 kHz. Moreover, from Figure 7, it can be found that the experimental and simulation results differ at frequencies ranging from 270 kHz to 430 kHz. The factors, such as material inhomogeneity, adhesive, crosstalk signal caused by equipment, could lead to the difference between experiments and simulations. However, as mentioned above, the signals outside the bandwidth range will be filtered out in the signal processing. In the 1–5 orientation, the experimental and the simulation results showed that the scattered waves were dominated by S0. However, there are nuances in the bandwidth of the STFT display. This is due to that the post-excitation ringing or resonance in PZT cannot be simulated effectively [34]. In contrast, for 3–7 orientation, the scattered waves were dominated by A0 mode.

**Figure 8:**
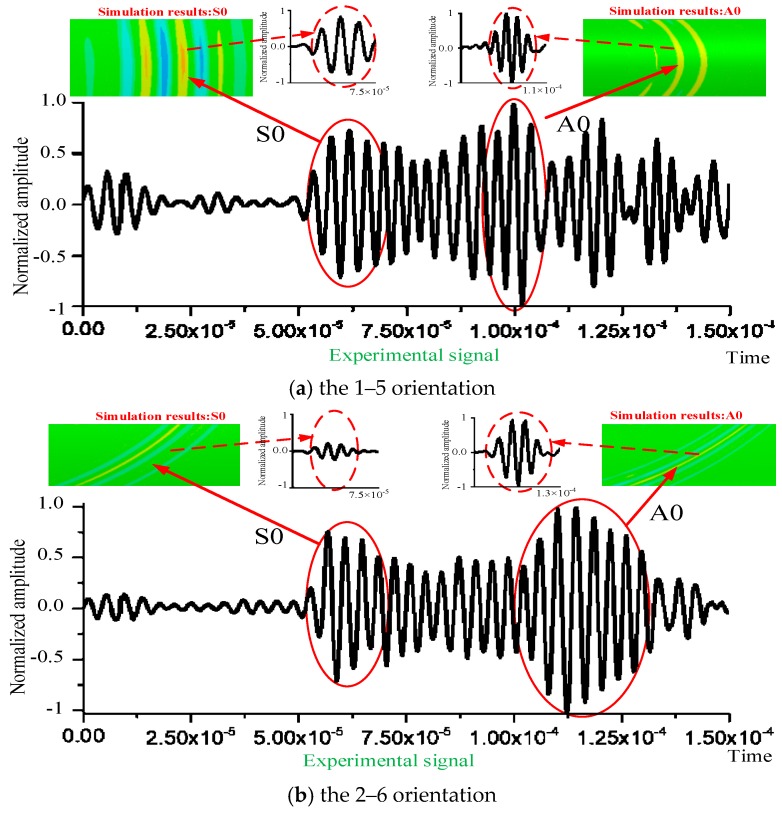

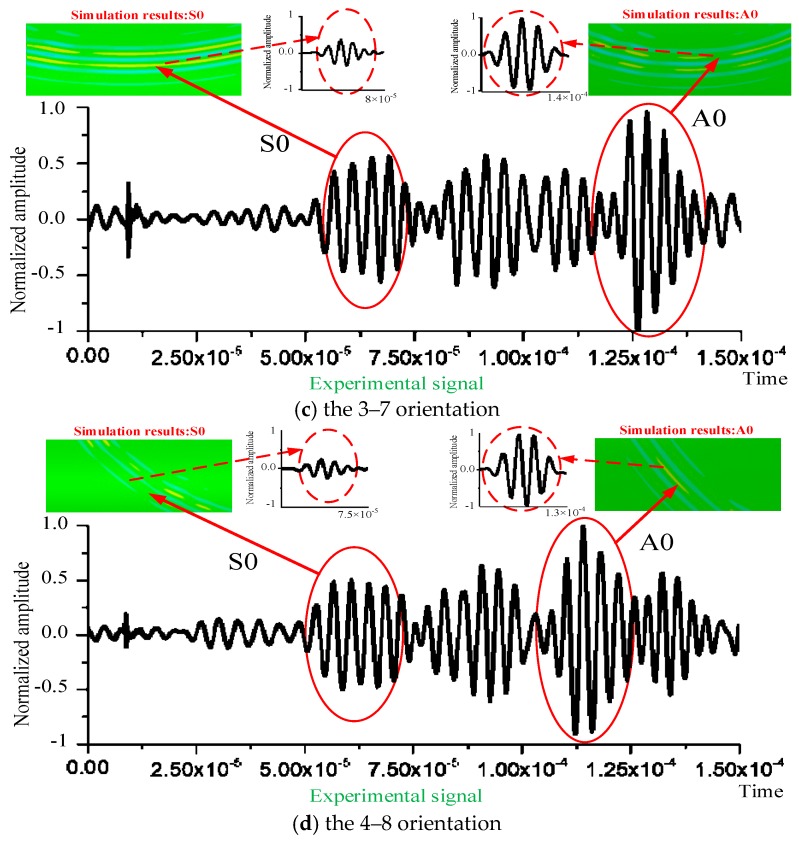
Comparisons between the simulation and the experimental results.

Figure 8 shows the comparison between the simulation results and the experimental signals of the curved plates. Then the wave packets of the two modes in the experimental signal are marked in the figure, and the images and signals of A0/S0 modes corresponding to the experimental signals of the simulation results are also displayed. Comparing the waveform signals, it can be found that the experimental and simulation results were consistent, and the amplitude distributions of the A0 and S0 modes were essentially the same. The continuous signal trail between S0 and A0 modes was caused by dispersion, and the two modes can be determined in the time domain by the wave velocity and the propagation distance. Moreover, the noise observed at the beginning of the recording time is the signal crosstalk caused by the instrument itself, which is unavoidable. This further indicates that the notch in the curved plates can be analyzed by A0/S0 mode features in the following work.

### 4.2. Notch Depth Effect on Guide Wave Characteristics

**Figure 9:**
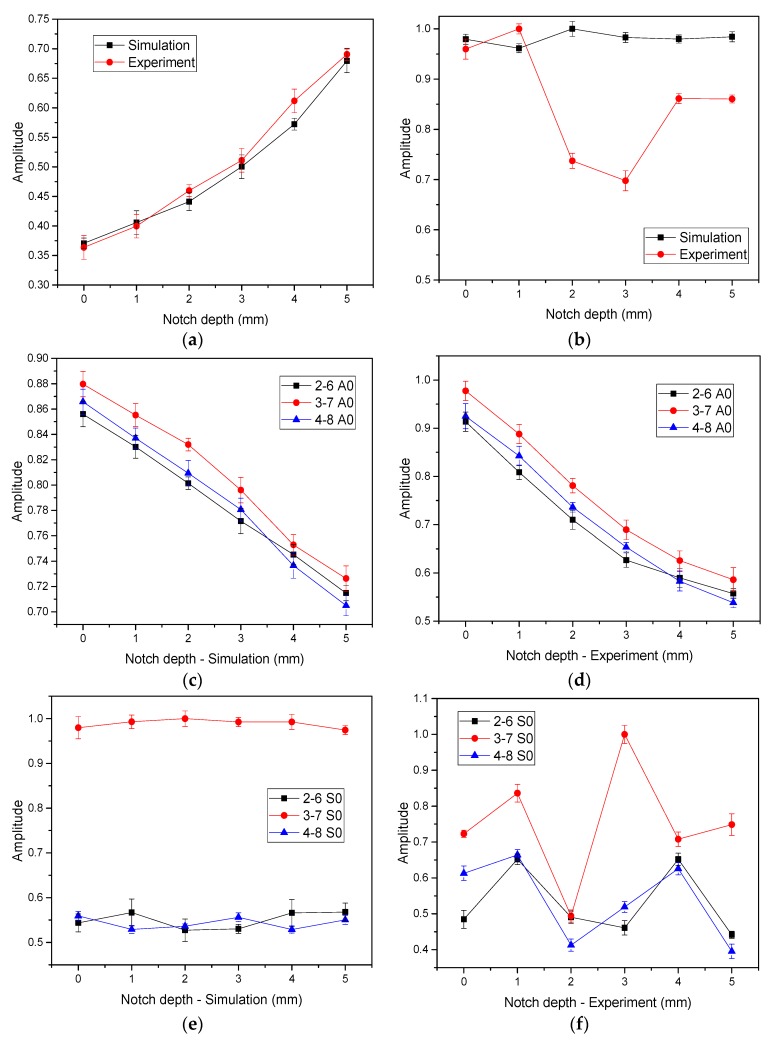
Amplitude of A0 and S0 signals varies with the notch depth. (**a**) S0 mode in the 1–5 orientation, (**b**) A0 mode in the 1–5 orientation, (**c**) A0 mode of simulation signals in the 2–6, 3–7, 4–8 orientation, (**d**) A0 mode of experiment signals in the 2–6, 3–7, 4–8 orientation, (**e**) S0 mode of simulation signals in the 2–6, 3–7, 4–8 orientation, and (**f**) S0 mode of experiment signals in the 2–6, 3–7, 4–8 orientation.

A0 and S0 wave modes were determined according to the velocities of the direct wave. The arrival time of the first S0 wave packet was 0.06 ms, and it is 0.125 ms of the A0 wave packet, which was consistent with the theoretical velocity in the dispersion curve. Figure 9 shows the normalized A0/S0 amplitude. As can be seen from Figure 9a,b, the amplitude of S0 mode in the 1–5 orientation increased with the increasing of notch depth, while no obvious change was found in A0 mode versus notch depth. The interaction between the guided wave signals and the notch led to the mode conversion, which led to the increase in the composition of S0 mode [31]. In the 2–6, 3–7, 4–8 orientations, the amplitudes of A0 mode decreased with the increasing of notch depth, as shown in Figure 9c,d. However, no obvious change was found in S0 mode versus notch depth as shown in Figure 9e,f. This difference was caused by the propagation orientations of guided waves. In case of 1–5, the guided wave propagated in the orientation of axial, the symmetric mode S0 was sensitive to notch damages. In the other three orientations, there was an angle between the guide wave propagation orientation and the axial orientation, and the existence of the curved surface produced a certain wave enhancement effect on the A0 mode [35]. With the increasing of notch depth, the wave enhancement effect was weakening, and the amplitude of A0 mode was decreased. The discrepancy in the experimental and numerical results should be caused by the mode conversion of the interaction between the guided wave signals and the notches, and the influence of factors such as crosstalk signals, adhesive had caused further difference.

### 4.3. Notch Orientation Effect on Guided Wave Characteristics

**Figure 10:**
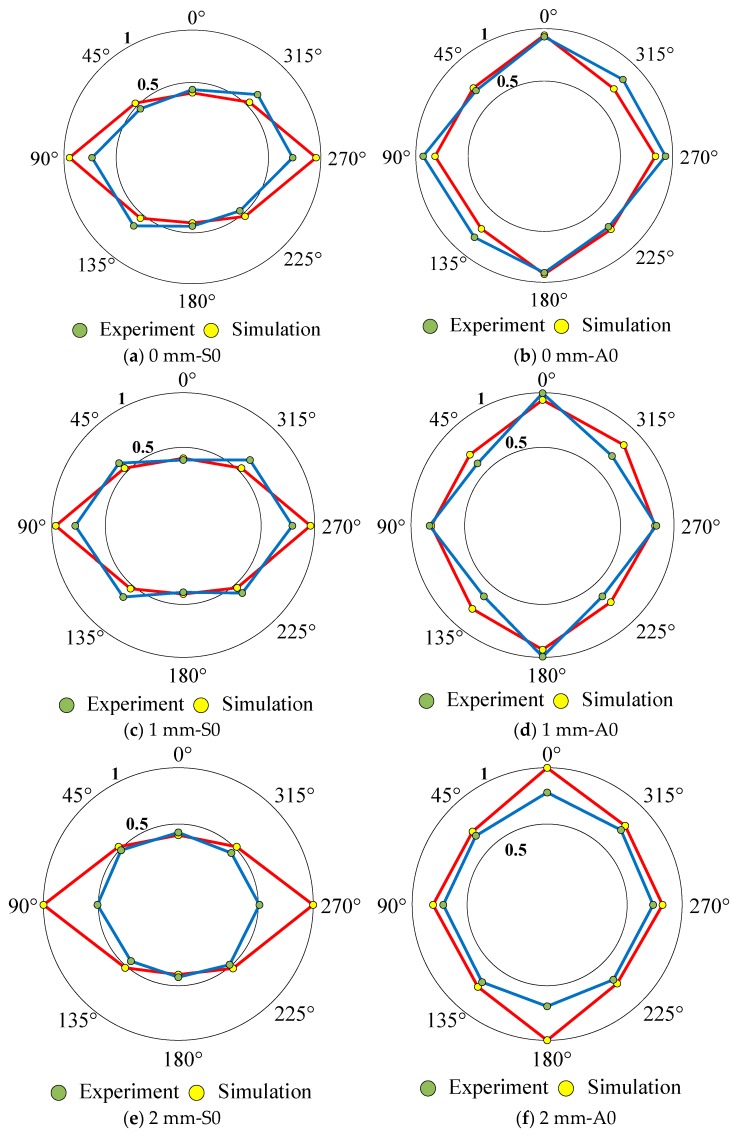

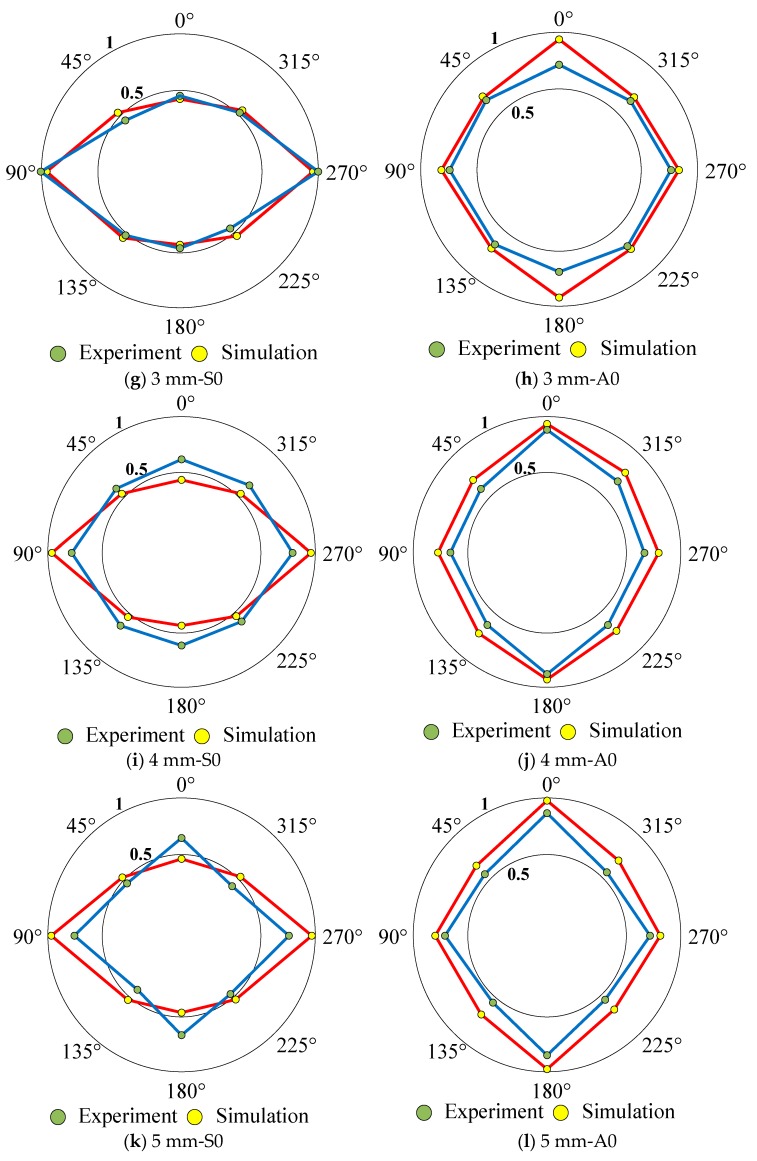
The directivity diagrams of curved plate under S0 and A0 modes.

In order to verify notch orientation effect on guided wave characteristics, the directivity diagrams were considered. The normalized amplitudes in different orientations were extracted, and the variation of the amplitude with the orientation can be described as shown in Figure 10. It can be seen that the simulated directivity curves were similar to the experimental curves, and the maximum/minimum A0/S0 amplitude were the same. The amplitude in 3–7 orientation was the largest for S0 mode, while it was the smallest in the 1–5 orientation. In terms of the A0 mode, the amplitude in 1–5 orientation was the largest, while in 3–7 orientation was relatively small. This was mainly caused by the angle between the sensors and the notch. The S0 mode amplitude in 1–5 orientation increased with the increasing of notch depth, while the A0 mode amplitudes in the 2–6, 3–7, 4–8 orientations decreased with the increasing of notch depth. As discussed above, this phenomenon indicates that the amplitude of S0/A0 modes are related to the notch depth.

### 4.4. Curved Plate Radius Effect on Guided Wave Characteristics

**Figure 11:**
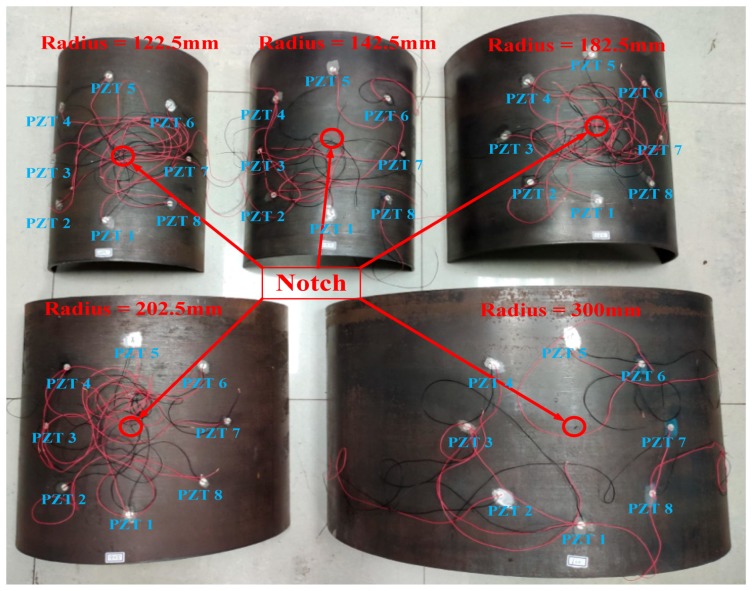
Curved plates with different radius.

**Figure 12:**
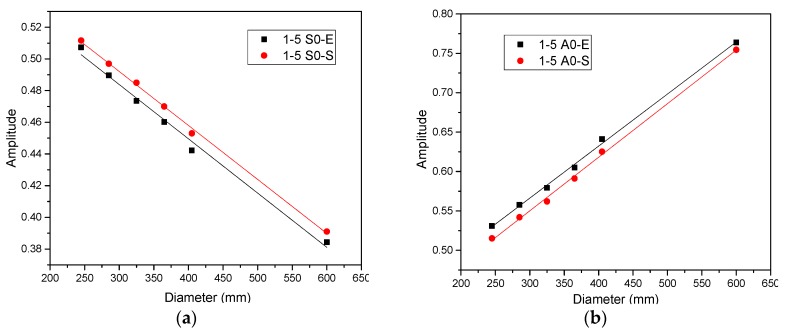

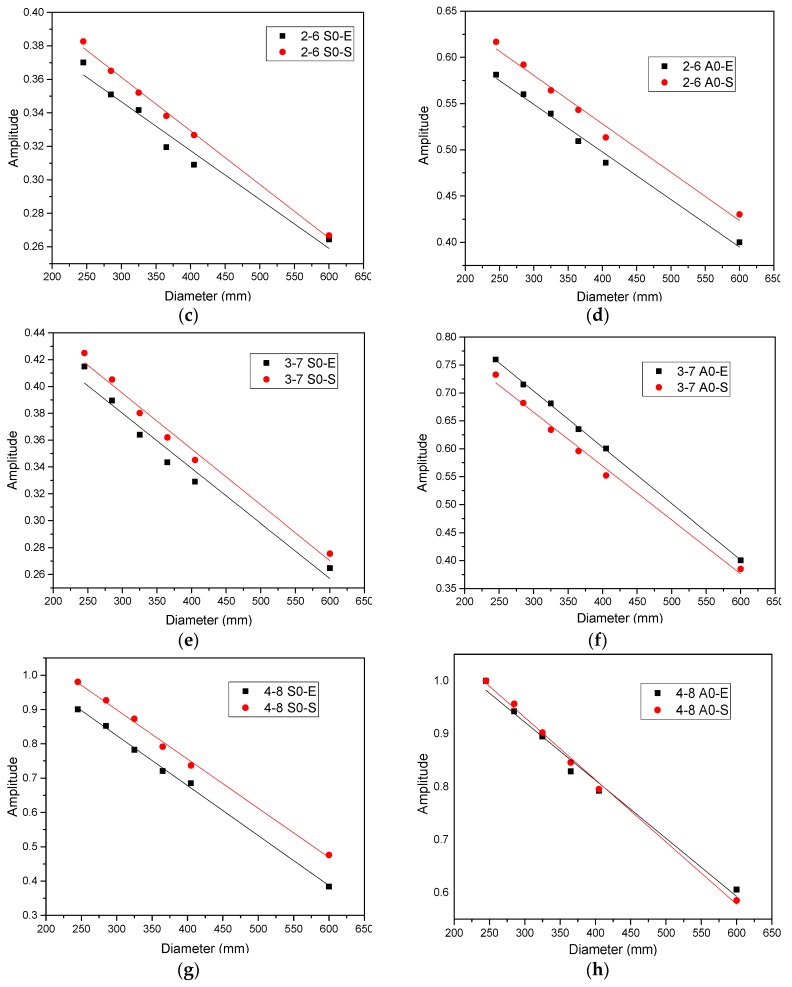
Amplitude of A0 and S0 varies with radius. (**a**) S0 mode in the 1–5 orientation, (**b**) A0 mode in the 1–5 orientation, (**c**) S0 mode in the 2–6 orientation, (**d**) A0 mode in the 2–6 orientation, (**e**) S0 mode in the 3–7 orientation, (**f**) A0 mode in the 3–7 orientation, (**g**) S0 mode in the 4–8 orientation, and (**h**) A0 mode in the 4–8 orientation.

Five curved plates with different radius were considered additionally to study the effect of radius on guided wave characteristics, as shown in Figure 11. The radii of the curved plates varied from 122.5 mm to 300 mm with the wall thickness of 5 mm, as the detailed parameters were shown in Table 2. The positions of notches and PZTs were the same as that in Figure 5, and the distance from notch to each PZTs was 125 mm. The damage scatter signals captured for the guided wave propagation path 1–5, 2–6, 3–7, and 4–8 were analyzed in Figure 12, respectively. The generation and acquisition of Lamb waves were fulfilled using online structural health monitoring system in the experiment, and the experimental signals were verified by simulation. To this end, the damaged curved plates with different radius were simulated in according to experiment. For smaller vessels, high quality signals can be obtained by changing the PZT array arrangements, for example, the positioning accuracy can be improved by arranging sparse PZT array arrangements [21].

From Figure 12, one can see that the influences of the radius on the normalized A0/S0 amplitude of each curved plate in four orientations are obvious. The variation of the A0/S0 amplitude in the curved plate with the radius from 122.5 mm to 202.5 mm can be obtained and, subsequently, the variation trend of the guided wave characteristics was verified by the curved plate with the radius of 300 mm. We would like to emphasize that Figure 12a,c–h show that the decrease in the other normalized A0/S0 amplitudes with radius are reasonably linear for the guided wave propagation path 1–5, 2–6, 3–7, and 4–8, and thus the agreement with the FEM results were excellent. It’s indicated that the increase of the ratio of radius to thickness leads to the increasing of wave velocity [19], which leads to the energy decay faster and the decay linear. It is also observed that the normalized A0 amplitude in the 1–5 orientation increases with the radius increases. As a result of the attenuation of the radius effect, the energy decay of the guided wave to be tiny, and the amplitude of A0 increases on account of the mode transformation.

### 4.5. Notch Localization in 30CrMo Steel Curved Plates by Ellipse-Based Algorithm

**Figure 13:**
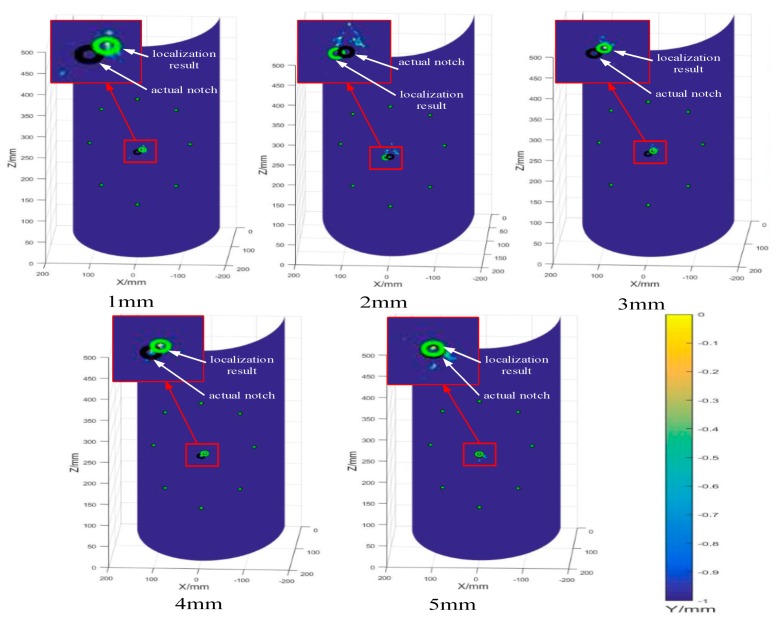
Damage localization results.

An ellipse-based algorithm was adopted to locate the notches in 30CrMo steel curved plates, as shown in Figure 13. Guided wave signals on the curved plates were obtained through the online structural health monitoring system. Based on the above conclusion, S0 wave packet was mainly considered for guided wave signals in the 1–5 orientation, while A0 wave packets were considered in the other three orientations. In Figure 13, eight PZT sensors are shown in a circular array. The black marks on the curved plates represent the actual notches, and the green marks indicate the localization result.

The results based on different modes are relatively accurate, and the localization errors are listed in Table 3.

The errors in the table are determined by Equation (12), as follows:

(12) $$Error(\%)=\frac{Predicted\;position-Actual\;position}{Actual\;position}\times 100\%$$

The average errors are the average of the circumferential errors and the axial errors. In the table, the localization errors show that the proposed localization method is feasible and has high localization accuracy. As the notch depth decreases, the localization error increases. For 5 mm-depth notch, the localization error was merely 0.005%, while the localization error corresponding to 1 mm-depth notch reached to 1.47%. The ellipse-based algorithm can locate the notch damage in the curved plate well, and it can be extended to locate the damage in pressure vessel.

**Figure 14:**
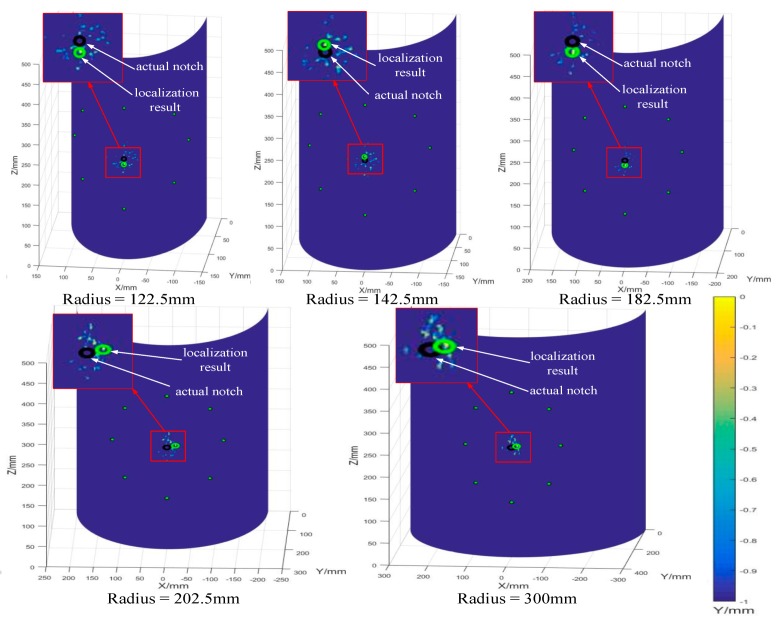
Damage localization results of curved plates with different radius.

According to the notch localization algorithm in curved plates, all the localization results of notched plates with different radius can be obtained, as shown in Figure 14. The positions of the PZTs, the actual notch and the localization results were given in the Figure. As can be seen in Figure 14, the localization results using the developed algorithm has good agreement with the actual notch positions, and the localization errors are listed in Table 4. It is observed that the localization method has high localization accuracy and good applicability to curved plates with different radius, and the maximum localization error was merely 1.20%.

## 5. Conclusions

The aim of this paper was to investigate the notch orientation and depth effect on the guided wave propagation behavior in 30CrMo steel curved plates with different radius. An ellipse-based algorithm and an online monitoring system were established to locate the notches in the curved plates. The following conclusions can be drawn:

As for the scatter wave in curved plate with 5 mm length notch, the STFT results showed that the A0 and S0 modes can be well separated at the frequency of 210 kHz. The experimental and simulation results match well, and A0/S0 amplitude distributions are essentially the same.

Diagrams showed the amplitude in the 3–7 orientation was the largest for S0 mode, and the amplitude of S0 mode in the 1–5 orientation increased with the increasing of notch depth. The amplitude of A0 mode in 1–5 orientation was the largest, while it decreased with the increasing of notch depth in 2–6, 3–7, 4–8 orientations.

Radius of curved plates has significant influences on the wave characteristics. The normalized A0 amplitude in the 1–5 orientation increases with the increasing of radius. As the increase of radius leads to the faster energy decay, the other normalized A0/S0 amplitudes are linear reduction for the guided wave propagating path 1–5, 2–6, 3–7, and 4–8.

For locating damage in the curved plates, the localization errors increase from 0.005% to 1.47% with the notch depth decreasing from 5 mm to 1 mm, while the maximum positioning error is 1.20% for curved plates with different radius. The results indicated that the ellipse-based algorithm has high positioning accuracy.

## Figures and Tables

**Table 1:** Parameters of the 30CrMo steel curved plate.

Item	Density/kg/m^3^	Modulus/MPa	Poisson’s Ratio	Diameter/mm	Thickness/mm
**Value**	7850	211,000	0.279	325	5

**Table 2:** Parameters of curved plates with different radius.

Radius/mm	Material	Length/mm	Thickness/mm	Notch Depth/mm
122.5, 142.5, 162.5, 182.5, 202.5, 300	30CrMo steel	500	5	5

**Table 3:** Localization errors of curved plates with different notch depths.

Notch Depth/mm	Circumferential Error	Axial Error	Average Error
1	1.51%	1.43%	1.47%
2	1.05%	0.85%	0.95%
3	0.90%	0.90%	0.90%
4	0.50%	0.60%	0.55%
5	0.01%	0	0.005%

**Table 4:** Localization errors of curved plates with different radius.

Radius/mm	Circumferential Error	Axial Error	Average Error
122.5	2.02%	0.38%	1.20%
142.5	0.95%	0.73%	0.84%
182.5	1.37%	0.69%	1.03%
202.5	0.67%	1.16%	0.92%
300	0.36%	0.86%	0.61%
